# A D-Shaped SPR-Based PCF Sensor with an Extremely High-Amplitude Sensitivity for Measuring the Refractive Index

**DOI:** 10.3390/mi14071295

**Published:** 2023-06-24

**Authors:** Wangyoyo Li, Yu Chen, Jianjie Xu, Menglin Jiang, Hui Zou

**Affiliations:** 1 Bell Honors School, Nanjing University of Posts and Telecommunications, Nanjing 210023, China; b20040713@njupt.edu.cn; 2 Portland Institute, Nanjing University of Posts and Telecommunications, Nanjing 210023, China; 3 College of Electronic and Optical Engineering & College of Flexible Electronics (Future Technology), Nanjing University of Posts and Telecommunications, Nanjing 210023, China

**Keywords:** photonic crystal fibre, surface plasmon resonance, refractive index sensor

## Abstract

In this work, a new D-shaped photonic crystal fibre sensor structure based on surface plasmon resonance (SPR) is purposed for measurement of analyte refractive index (RI). In this design, a silica cylinder is polished into a D-shaped silica material structure with a flattened surface where two Au nanowires are coated. Some air holes are omitted to form the core mode region. With the implementation of gold nanowires and a core region for the exciting SPR effect with variate physical values, analyte RI can be measured with a wavelength coverage from 850 to 1350nm. The numerical simulation shows the maximum wavelength sensitivity of the purposed design achieves 19,600nm/RIU with an RI coverage from 1.37 to 1.42. Moreover, the sensor has a tremendous amplitude sensitivity and the maximum absolute value is about $2300\;{\mathrm{RIU}}^{-1}$. Benefiting from these outstanding performance, the purposed structure can be given priority when it is applied in biomedical detecting and environmental assessment science.

## 1. Introduction

Surface plasmon resonance (SPR) is a collective oscillation of conduction band electrons which are interacting with the oscillating electric field of the incident light to cause resonance [1]. It is an optical effect where the incident light with an appropriate incidence angle brings photons to the surface of metal and plasmonic materials, the surface-located electrons interact and are excited. Then the electrons propagate parallel to the surface and produce energetic plasmon electrons through non-radiative excitation [2,3]. When the SPR effect occurs, a tiny deviation in the analyte refractive index (RI) or other variables of the propagation medium and multi-physics system will change the phase-matching condition and further hinders the SPR excitation [4,5], making it play a dominant role in real-time interaction sensing of biomolecular binding events [6] and feasible for analyte measurements and multi-physics system sensing, especially medical reagent testing services in the global public health crisis [7,8,9]. In recent years, with extensive research on photonic crystal fibres (PCFs) [10], findings have concluded that PCF is an excellent environment for the SPR effect to be excited because of its distinctive optical properties and a designable structure with different geometries and materials to choose from [11,12,13,14]. A great deal of work has been dedicated to propose SPR-based PCF sensors to achieve favourable results in measurement and detecting in multi-physics systems such as the analyte RI [15], temperature [16], magnetic field [17], salinity [18], etc.

With development of the structural design and manufacturing process, a multitude of works have focused on SPR-based PCF sensors of various topological geometries along with different plasmonic materials. In 2017, Hoque et al. proposed a point-of-care diagnosis medical device using a unique SPR-based PCF structure which applied a U-grooved with a selective coating to achieve remote and biosensing applications with extreme scenarios. In this work, the proposed structure achieved maximum amplitude sensitivity $S_{a}$ and wavelength sensitivity $S_{n}$ value about 1189 ${\mathrm{RIU}}^{-1}$ and 12,500 nm/RIU, respectively, leading to an outstanding resolution of $8\times 10^{-6}$ RIU. Moreover, the proposed structure had a large analyte RI coverage from 1.29 to 1.40 [19]. One year later in 2018, Hasan et al. achieved a high sensitivity and appropriate linearity-characteristics performance by introducing a dual-polarized spiral-shaped PCF. In this work, an $S_{n}$ value of 4600 and 4300 nm/RIU in both *x*- and *y*-polarized modes with analyte RI set as 1.37 was achieved. Furthermore, with an analyte RI coverage from 1.33 to 1.38, the maximum $S_{a}$ values of 371.5 ${\mathrm{RIU}}^{-1}$ and 420.4 ${\mathrm{RIU}}^{-1}$ were achieved in both *x*- and *y*-polarized modes, respectively, [20]. In 2020, Tong et al. proposed a high-sensitivity biosensor structure by applying a metal nanowire array to a polished D-shaped dual-core PCF. In this work, with an analyte RI coverage chosen from 1.32 to 1.38, the proposed sensor obtained a maximum $S_{n}$ value of 16,000 nm/RIU [21]. In 2022, Chao et al. proposed a simple elliptical air hole PCF sensor comprising an analyte channel on the structure’s surface along with a flat surface and external surface coated with gold [22]. Moreover, Chao et al. proposed in 2023 an extraordinary PCF temperature sensor which utilized a flat, metal-coated trapezoidal surface. This structure provided a bimodal feature of both an effective refractive index and confinement loss [23].

However, measurement of a single parameter cannot meet the requirements in complicated real-life application scenarios. Moreover, in multi-physics systems, the optimization of one single parameter cannot achieve the final optimal outcome. Therefore, a host of multi-parameter sensors are proposed to satisfactorily resolve the problem above in contemporary research. In 2022, Wang et al. proposed a sensor structure capable of both magnetic field and temperature detection. With two elliptical channels coated with gold/${\mathrm{Ta}}_{2}O_{5}$ film and filled with magnetic fluid/polydimethylsiloxane utilized on both sides of the fibre core area, the sensor achieved the sensing mechanism. It showed that with a magnetic field intensity coverage from 50 to 130 Oe, the magnetic field sensitivities of the two channels could reach 65 and 0 pm/Oe, respectively. Moreover, with a temperature coverage from of 17.5 to $27.5{\;}^{\circ }C$, the temperature sensitivities of two channels could achieve 520 and 2360 $\mathrm{pm}{/}^{\circ }C$, respectively. However, the sensor did not obtain a simultaneous measurement of the temperature and magnetic field, demonstrating the sensor had a correlated dual-parameter sensing performance [24].

Some other bottlenecks are that the sensor may encounter serious crosstalk problems between different sensing parameters and the sensor is incapable of simultaneously measuring multiple variables. Up till now, some works have overcome the difficulties and achieved simultaneous sensing. In 2023, Su et al. proposed a multi-channel SPR-based PCF structure utilizing both *x*- or *y*-polarized modes along with multiple plasmonic materials in order to efficiently lower the correlated interference between different channels and make the SPR effect further excited and strengthened. Therefore, the sensor simultaneously attained measurement in four channels along with low interference. With the fibre polished into four channels and metal materials gold/silver and plasmonic materials ${\mathrm{TiO}}_{2}$/${\mathrm{Ta}}_{2}O_{5}$/graphene were chosen as coatings, the sensor reached the design requirements with four channels detecting analyte RI coverage from 1.34 to 1.42. It was shown that the four channels of the structure had maximum $S_{n}$ values of 49,800, 49,000, 35,900, and 36,800 nm/RIU, respectively, [25]. In different topological structures the sensing performance and operating wavelength range were desperate. Previous works have reached great wavelength sensitivity with small amplitude sensitivity. Therefore, it is of great importance that a structure with both great wavelength sensitivity and amplitude sensitivity is proposed.

In this work, a new D-shaped PCF sensor structure based on SPR is proposed for the measurement of analyte RI with an extremely high $S_{a}$ value. The sensing performance of structure in this work is investigated through numerical simulation with help of finite element method (FEM). With the implementation of two Au nanowires along with core regions for exciting the SPR effect with variate physical values, the RI value can be measured with a wavelength coverage from 850 to 1350nm. Furthermore, with a significant red-shift in the confinement loss (CL) curve when the analyte RI value changes from 1.41 to 1.42, the structure obtains an extreme high $S_{a}$ value. The numerical simulation shows that the maximum $S_{n}$ value of the purposed design achieves 19,600nm/RIU with a RI coverage from 1.37 to 1.42 and a maximum absolute amplitude sensitivity of $2300\;{\mathrm{RIU}}^{-1}$. Benefiting from this outstanding performance, the purposed structure can be given priority when it is applied in biomedical detecting and environmental assessment science.

## 2. Design and Analysis of the Structure

**Figure 1:**
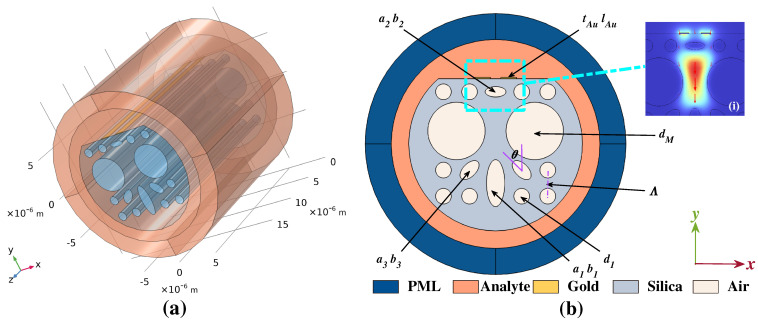
(**a**) A 3D view of the proposed structure. (**b**) A 2D schematic of the proposed structure. Sub-figure (**i**) shows the mode field distribution of the core mode.

Figure 1a and Figure 1b show a 3D view and 2D schematic of the proposed D-shaped sensor structure, respectively. The sub-figure (i) shows the mode field distribution of the core mode, applied for refractive index (RI) measurements. The proposed fibre has an area comprising a 5×5 air hole array arranged in a square lattice. The lattice spacing of the array is denoted by Λ and the basic small air holes have a radius denoted by $d_{1}$. Some basic small air holes are replaced with elliptical holes and big air holes to strengthen the SPR. To ensure the SPR effect successful excitation, a part of the fibre background material is polished with an appropriate distance to the centre to form a D-shaped structure and the flattened plane is coated with two gold nanowires with the thickness and length donated by $t_{Au}$ and $l_{Au}$, respectively. In the central region of the silica, two basic small air holes are omitted for the sake of forming a core mode area, four adjacent downward air holes are replaced with one large and two basic small elliptical holes pointing to the core region to strengthen the SPR effect, the major and minor semi-axis of one big and two small elliptical air holes are represented as $a_{1}$, $b_{1}$ and $a_{3}$, $b_{3}$, respectively. During fabrication, tolerance may exist when controlling the elliptical air holes for pointing to the core region, the related effects of the deviation are discussed in the following section. Meanwhile, to further strengthen the intensity of the core mode area when the SPR effect occurs, the upward basic small air hole is replaced with a horizontal ellipse with the major and minor semi-axis represented as $a_{2}$ and $b_{2}$, respectively. Moreover, eight basic small air holes on the left and right side of the core mode area are replaced into big air holes with radius $d_{M}$ to compress the core region and improve sensor performance. A liquid analyte is introduced on the external part of the fibre.

In the numerical simulation, this work uses finite element method (FEM) to solve an eigenmode. Furthermore, a perfectly matched layer (PML) with a thickness chosen as $t_{PML}=2.0\;$ μm is placed outside the analyte area to truncate the region beyond the fibre structure and limit the computational area by absorbing all radiation energy [26,27].

**Figure 2:**
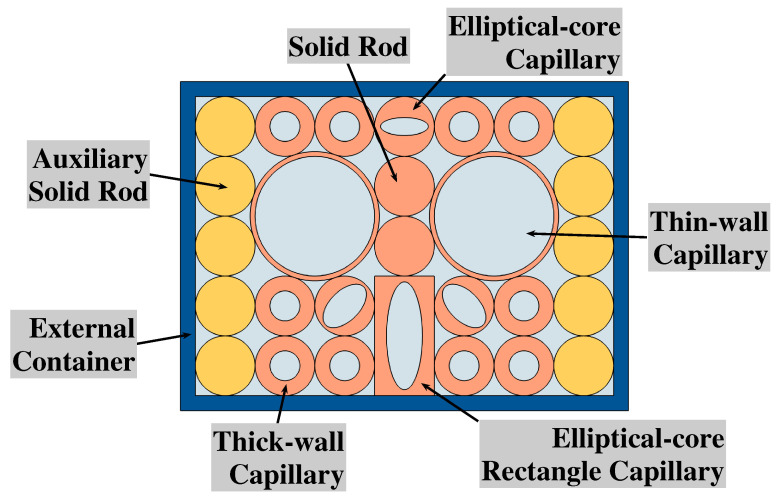
A 2D schematic of the proposed sensor structure in the fabrication process.

Figure 2 demonstrates the 2D schematic of the proposed sensor structure in the fabrication process. In the process, we utilized an external container to gather the different types of capillaries and rods in the start-up stage. In this paper, thick-wall, thin-wall, and elliptical-core fibres are applied to form the small hole, big hole and basic elliptical core, respectively. Two solid rods were used to form the core area. An elliptical-core rectangle capillary was utilized in order to form the big elliptical-core beneath the core area. Moreover, ten auxiliary solid rods, as seen in gold in Figure 2, were designed for fixed position of the proposed structure part. After bringing these capillaries and rods together in a rectangular external container, liquid silica is allowed to infiltrate the space among these structures and the start-up structure is made. Then the auxiliary solid rods are removed and the remaining part is put into a circular external container, after pouring the fused silica, the final sensor structure is thereby made.

Fused silica is applied as the fibre material for its superior properties and performance, with a material RI which can be calculated by the third-order Sellmeier Equation (1) [28], where λ denotes the wavelength in free-space.

(1) $$n_{Silica}^{2}=1+\frac{0.6961663\lambda^{2}}{\lambda^{2}-0.0684043^{2}}+\frac{0.4079426\lambda^{2}}{\lambda^{2}-0.1162414^{2}}+\frac{0.897479\lambda^{2}}{\lambda^{2}-9.896161^{2}}$$

Two gold nanowires, similar to the other metal materials, are used to excite the SPR effect. The dispersion of the gold nanowires can be easily obtained via the Lorentz–Drude model expressed in the following Equation [29,30].

(2) $$\varepsilon_{m}=\varepsilon_{\infty }-\frac{\omega_{D}^{2}}{\omega (\omega -j\gamma_{D})}-\frac{\Delta \varepsilon \cdot \Omega_{L}^{2}}{\left(\omega^{2}-\Omega_{L}^{2}\right)-j\Gamma_{L}\omega }$$

where $\varepsilon_{\infty }=5.9673$ denotes the dielectric constant with a ultra-high frequency, and ω=2πc/λ stands for the angular frequency of the light propagating through the proposed fibre. $\omega_{D}$ and $\gamma_{D}$ represent the plasma and damping frequency, respectively. Δε=1.09 is the weight factor. $\Omega_{L}$ and $\Gamma_{L}$ are the frequency and spectral width of the Lorentz oscillator, respectively, where $\Omega_{L}/2\pi =650.07\;\mathrm{THz}$ and $\Gamma_{L}/2\pi =104.86\;\mathrm{THz}$ in angular frequency.

It is generally acknowledged that the SPR-based PCFs work owing to the interactions between electrons on the surface of the plasmonic material along with the evanescent field [31,32]. The confinement loss (CL) is a good metric representing the loss in propagation, which can be calculated using Equation (2) [33,34], where $n_{eff}$ denotes the effective RI of the core mode. The unit of CL value is dB/cm.

(3) $$CL=8.686\times \frac{2\pi }{\lambda }\times Im\left(n_{eff}\right)\times 10^{4}$$

In this article, utilizing two Au nanowires and core regions to excite the SPR effect with variable physical values, the RI value can be measured with a wavelength coverage from 850 to 1350nm. Furthermore, with a significant red-shift in the CL curve when the RI value changes between 1.41 and 1.42, the structure achieves an extreme high $S_{a}$ value.

**Figure 3:**
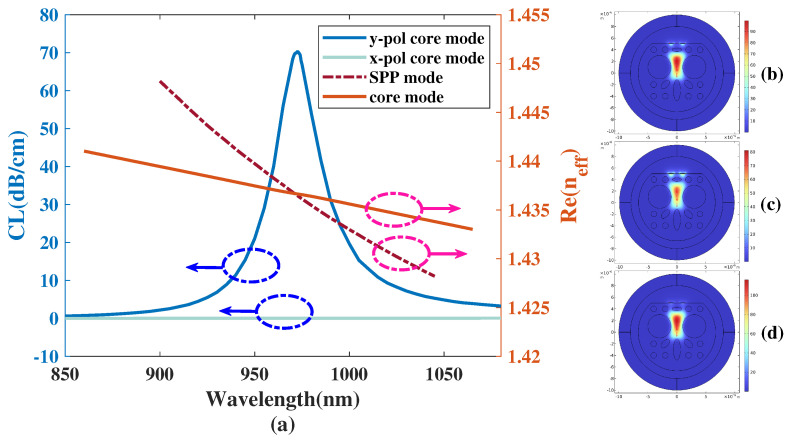
(**a**) The CL curve of both the *x*- and *y*-polarized mode and the real part of the effective RI of the core modes and SPP mode when the analyte RI is 1.40. (**b**−**d**) The mode field distributions of the simulated proposed structure with wavelengths of 950, 973, and 1050 nm, respectively.

Figure 3a illustrates the CL curve of both the *x*- and *y*-polarized modes and the real part of the effective RI of the core modes and SPP mode when the analyte RI is 1.40 with a list of structural parameters of the proposed design: $a_{1}=1.8\;$ μm, $b_{1}=0.6\;$ μm, $a_{2}=0.8\;$ μm, $b_{2}=0.3\;$ μm, $a_{3}=0.9\;$ μm, $b_{3}=0.5\;$ μm, $d_{1}=0.5\;$ μm, $d_{M}=2\;$ μm, $t_{Au}=60\;$ nm, $l_{Au}=1\;$ μm, Λ=2 μm. Furthermore, Figure 3b–d shows the mode field distributions of the proposed structure simulated with wavelength of 950, 973, 1050 nm, respectively. In Figure 3a, the core mode along with the SPP mode totally couple together in the area of the polished Au-coated plane when the wavelength is about 973 nm. The value of the CL curve is tiny in those wavelength regions where resonance almost does not occur. A reasonable explanation for this phenomenon is that the majority of energy is limited in the core region when the wavelength is in those non-resonant regions, as illustrated in Figure 3b,d. When SPR is fully excited with a resonant wavelength $\lambda_{SPR}$, the core mode couples along with the SPP mode and energy distribution changes from the core area to the surface of the flattened Au-coated plane, as illustrated in Figure 3c. At this moment, the phase-matching condition is achieved, as shown in the intersection between the orange solid curve and crimson dash–dot curve in Figure 3a. Meanwhile, the CL curve reaches a peak. Moreover, Figure 3a shows that on a dB-scale the CL value of the core mode in *y*-polarization is already much greater than the core mode in *x*-polarization, which is almost fluctuating around 0 dB/cm. This further illustrates the realization of the single-polarization sensing performance. Therefore, we select *y*-polarization for operation in this work.

The variation in the analyte RI impacts the effective RI in the region of the excited SPR effect, further changing the mode field distributions and the related $\lambda_{SPR}$ value. Eventually, the CL curve starts to red- or blue-shift and the peak value changes. Thus, it is of great significance to find an evaluation scheme to measure the correlation between the changing parameters and the affected sensor performance. In this scenario, the correlation is appropriate to measure the variation of the CL curve in conjunction with the analyte RI. Therefore, there are two good metrics: RI sensitivity ($S_{n}$) and amplitude sensitivity ($S_{a}$), which are defined in the equations below [35],

(4) $$S_{n}=\Delta \lambda_{SPR}/\Delta n_{a}$$

(5) $$S_{a}=-\left(\Delta CL/\Delta n_{a}\right)/CL_{initial}$$

where $\Delta \lambda_{SPR}$ represents the change in the resonant wavelength value, while $\Delta n_{a}$ stands for the RI variation. ΔCL represents the change in the confinement loss and $CL_{initial}$ denotes the initial confinement loss. The unit of $S_{n}$ is in nm/RIU and the unit of $S_{a}$ is in ${\mathrm{RIU}}^{-1}$.

Another important metric in relation to the performance of the proposed sensor is the figure of merit, focusing on the full width at half maximum (FWHM) value. This is defined by the equation below [36],

(6) $$FOM=S_{n}/\Delta \lambda_{1/2}$$

where $\Delta \lambda_{1/2}$ denotes the FWHM.

## 3. Simulation Results and Discussion

**Figure 4:**
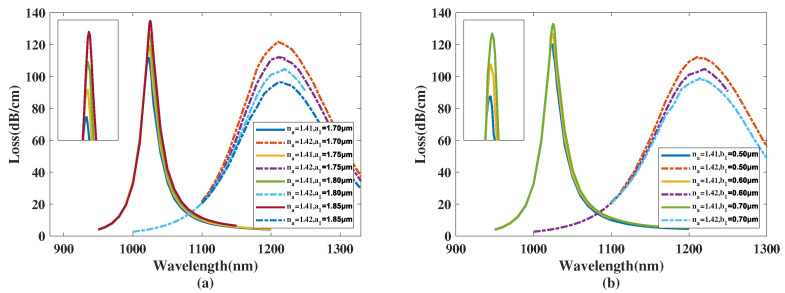
(**a**) The CL curve of the *y*-polarized mode with $a_{1}$ set as 1.70, 1.75, 1.80, 1.85 μm, and an analyte RI of 1.41 or 1.42. (**b**) The CL curve of the core mode with $b_{1}$ chosen as 0.50, 0.60, 0.70 μm, and an analyte RI of 1.41 or 1.42.

Figure 4a shows the CL curve of the *y*-polarized mode with $a_{1}$ chosen as 1.70, 1.75, 1.80, 1.85 μm, and the analyte RI is set as 1.41 and 1.42. From Figure 4a, with $a_{1}$ increasing from 1.70 to 1.85 μm, the $\lambda_{SPR}$ value of the CL spectra with analyte RI 1.41 starts to visibly red-shift and the peak value of the CL curve gradually increases. The resonance wavelength of the CL spectra with analyte RI 1.42 is almost constant and the peak value of the CL curve exhibits a noticeable decrease. Furthermore, when making a comparison among the CL peak values and related envelopes, it can be further inferred that with an increasing $a_{1}$, the envelope of the CL spectra associated with the changing analyte RI appears to blue-shift. A reasonable explanation for this phenomenon is when $a_{1}$ increases, the *y*-polarized mode region is compressed, causing the intensity of the envelope peak to visibly increase. However, the big elliptical hole, which has its major-axis denoted by $a_{1}$, has a long distance to the gold nanowires. So the variation in $a_{1}$ has an insignificant effect to the phase-matching condition, and thus the change has almost no impact on the resonance wavelength and RI sensitivity. In accordance with Equation (4), the maximum $S_{n}$ values achieved were 18,750, 18,850, 19,600, 18,800 nm/RIU with $a_{1}$ chosen as 1.70, 1.75, 1.80, 1.85 μm, respectively. The calculated $S_{n}$ values of the proposed structure firstly increase and then decrease with $a_{1}$ changing from 1.70 to 1.85 μm, reaching a maximum near $a_{1}=1.80\;$ μm. In accordance with Equation (5), the maximum $S_{a}$ values achieved were 2945, 2705, 2517.5, and 2311.25 $RIU^{-1}$ when $a_{1}$ was set as 1.70, 1.75, 1.80, 1.85 μm, respectively, and the analyte RI was set as 1.42, demonstrating that the $S_{a}$ value of the structure decreases when $a_{1}$ rises from 1.70 to 1.85 μm and always has a good sensing performance.

Figure 4b illustrates the CL curve of the *y*-polarized mode with $b_{1}$ chosen as 0.50, 0.60, 0.70 μm, and the analyte RI of 1.41 and 1.42. From Figure 4b, when the analyte RI is 1.41 and $b_{1}$ increases from 0.50 to 0.70 μm, the $\lambda_{SPR}$ value of the CL curve has a slight red-shift and the peak of the CL curve gradually increases. The resonance wavelength of the CL curve with an analyte RI of 1.42 is almost constant and the *CL* peak visibly decreases. Furthermore, when making a comparison among the CL peak values and related envelopes, it can be found that when the $b_{1}$ value increases, the envelope of the CL spectra in regard to changing the analyte RI appears to blue-shift slightly. A reasonable explanation for this phenomenon is that when $b_{1}$ increases, the *y*-polarized mode region is compressed. However, $b_{1}$ has a lesser effect on the core mode region compared to $a_{1}$; therefore, the intensity of the CL curve slightly increases and the $\lambda_{SPR}$ and $S_{n}$ values are almost constant. With the increase in the CL peak value with RI set as 1.41 and a decrease in the CL intensity when RI is 1.42, the related wavelength of the maximum value of the whole envelope of the CL curve gradually changes from 1200 to 1000 nm, which is a blue-shift of the envelope. In accordance with Equation (4), the maximum $S_{n}$ values achieved were 18,750, 19,600, 18,600 nm/RIU with $b_{1}$ chosen as 0.50, 0.60, 0.70 μm, respectively. The calculation results show the $S_{n}$ value of the proposed structure first increases and then decreases with $b_{1}$ changing from 0.50 to 0.70 μm, reaching a maximum near $b_{1}=0.60\;$ μm. In accordance with Equation (5), the maximum $S_{a}$ values achieved were 2705, 2517.5, and 2372 RIU${}^{-1}$ when $b_{1}$ was 0.50, 0.60, 0.70 μm, respectively, when the analyte RI was 1.42, illustrating that the $S_{a}$ value of the proposed structure diminishes when $b_{1}$ increases from 0.50 to 0.70 μm and always has a great sensing performance.

**Figure 5:**
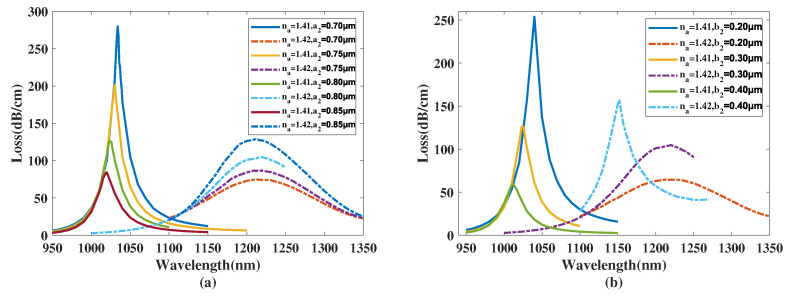
(**a**) The CL curve of the *y*-polarized mode with $a_{2}$ set as 0.70, 0.75, 0.80, 0.85 μm, and analyte RI as 1.41 and 1.42. (**b**) The CL curve of the core mode with $b_{2}$ set as 0.20, 0.30, and 0.40 μm, and analyte RI as 1.41 and 1.42.

Figure 5a demonstrates the CL curve of the *y*-polarized mode when $a_{2}$ was 0.70, 0.75, 0.80, 0.85 μm, and the analyte RI was chosen as 1.41 and 1.42. From Figure 5a, when the analyte RI was 1.41 and $a_{2}$ increases from 0.70 to 0.85 μm, the $\lambda_{SPR}$ value of the CL curve appears to red-shift with a noticeable trend and the CL peak value significantly falls. The resonance wavelength of the CL spectra with an analyte RI 1.42 has a slight blue-shift and the peak value of the CL curve visibly decreases. Furthermore, it is easy to infer from Figure 5a that the FWHM value of the CL curve almost remains intact when $a_{2}$ increases. Furthermore, when making a comparison among the CL peak values and related envelopes, it can be further deduced that when $a_{2}$ increases, the envelope of the CL spectra associated with the changing analyte RI appears to visibly red-shift. The main reason considered for this is that when $a_{2}$ increases, the channel linking the gold nanowires and the core mode area narrows, changing the distributions of the mode field and influences the *y*-polarized mode. In accordance with Equation (4), the maximum $S_{n}$ values achieved were 17,900, 18,900, 19,600, 19,100 nm/RIU with $a_{2}$ chosen as 0.70, 0.75, 0.80, 0.85 μm, respectively. The calculation results demonstrate that the $S_{n}$ value of the proposed structure first rises and then declines with the variation in $a_{2}$ from 0.70 to 0.85 μm, reaching a maximum near $a_{2}=0.80\;$ μm. In accordance with Equation (5), the maximum $S_{a}$ values achieved were 835.9, 1348.2, 2226.7, and 3577.143 RIU${}^{-1}$ when $a_{2}$ was set as 0.70, 0.75, 0.80, 0.85 μm, respectively, when the analyte RI was set as 1.42, illustrating that the amplitude sensitivity of the proposed structure significantly boosts when $a_{2}$ is changed from 0.70 to 0.85 μm, thus improving its sensing performance.

Figure 5b shows the CL curve of the *y*-polarized mode with $b_{2}$ chosen as 0.20, 0.30, and 0.40 μm, and the analyte RI as 1.41 and 1.42. From Figure 5b, when the analyte RI was 1.41 and $b_{2}$ increased from 0.20 to 0.40 μm, the $\lambda_{SPR}$ value of the CL curve has a visible blue-shift and the peak value of the CL curve falls significantly. The $\lambda_{SPR}$ value of the CL spectra with an analyte RI of 1.42 sees a significant blue-shift and the peak value of the CL curve appears to noticeable increase. Moreover, it is evident that the FWHM value of the CL spectra decreases when $b_{2}$ increases. Furthermore, when making a comparison among the CL peak values and related envelopes, it can be concluded that when the $b_{2}$ value increases, the envelope of the CL spectra in relation to the changing analyte RI appears the visibly red-shift. The main reason considered is that when $b_{2}$ increases, the region between the gold nanowires and silica area is compressed, leading to the changing the phase-matching condition, further affecting the resonance wavelength and peak intensity. In accordance with Equation (4), the maximum $S_{n}$ values achieved were 17,200, 19,600, 14,000 nm/RIU with $b_{2}$ chosen as 0.20, 0.30, 0.40 μm, respectively. The calculation results demonstrate that the $S_{n}$ value of the proposed structure first increases and then decreases with $b_{2}$ changing from 0.20 to 0.30 μm, reaching a maximum near $b_{2}=0.30\;$ μm. In accordance with Equation (5), the maximum $S_{a}$ values achieved was 488.37, 2226.7, 5874.8 RIU${}^{-1}$ when $b_{2}$ was set as 0.20, 0.30, 0.40 μm, respectively and the analyte RI was 1.42, illustrating that the $S_{a}$ value of the structure has a significant leap with $b_{2}$ increasing from 0.20 to 0.40 μm, thus optimizing its sensing performance.

**Figure 6:**
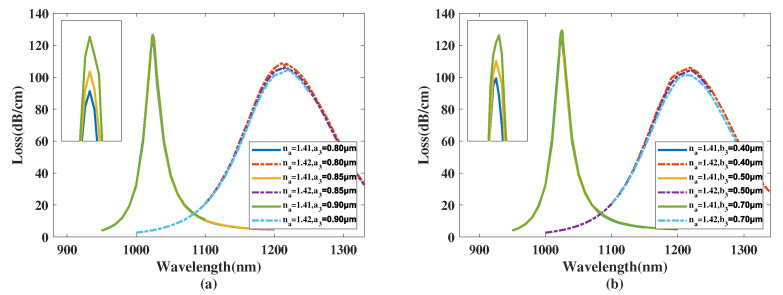
(**a**) The CL curve of the *y*-polarized mode with $a_{3}$ set as 0.80, 0.85, and 0.90 μm, and analyte RI as 1.41 and 1.42. (**b**) The CL curve of the core mode with $b_{3}$ set as 0.40, 0.50, and 0.70 μm, and the analyte RI is 1.41 and 1.42.

Figure 6a,b shows the CL curve of the *y*-polarized mode with $a_{3}$ chosen as 0.80, 0.85, 0.90 μm and $b_{3}$ set as 0.40, 0.50, 0.70 μm when the analyte RI is 1.41 and 1.42, respectively. From Figure 6a,b, when the analyte RI is chosen as 1.41 and 1.42 with $a_{3}$ and $b_{3}$ increasing, the $\lambda_{SPR}$ value of the CL spectra are almost constant and the CL peak value changes little. A reasonable explanation for this phenomenon is that the two elliptical small holes are further away from the two gold nanowires and the *y*-polarized mode region, so the variation has an almost negligible effect on the mode field distributions, thus having little impact on the *y*-polarized mode and sensing performance. In accordance with Equation (4), the maximum $S_{n}$ values achieved were 18,600, 19,300, 19,600 nm/RIU with $a_{3}$ set as 0.80, 0.85, 0.90 μm, respectively, and can reach 19,500, 19,600, 19,000 nm/RIU when $b_{3}$ is set as 0.40, 0.50, 0.70 μm, respectively. It can be concluded that the $S_{n}$ value of the proposed sensor reaches an optimal value near $a_{3}=0.90\;$ μm, $b_{3}=0.50\;$ μm. In accordance with Equation (5), the maximum $S_{a}$ value can reach about 2315.6, 2260, 2226.7 RIU${}^{-1}$ when $a_{3}$ is set as 0.80, 0.85, 0.90 μm, respectively, and can reach 2253.3, 2226.7, 2151.1 RIU${}^{-1}$ when $b_{3}$ is set as 0.40, 0.50, 0.70 μm, respectively, with an analyte RI chosen as 1.42. It can be deduced that the maximum $S_{a}$ value of the sensor has negligible change along with variation in $a_{3}$ and $b_{3}$, but the structure can always achieve an outstanding sensing performance.

Another factor which must considered is that in the fabrication process the small elliptical air holes may deviate from pointing towards the core area. In this work, θ is used to describe the angle of the direction of these elliptical holes and the vertical axis. In ideal condition, $\theta =45^{\circ }$.

**Figure 7:**
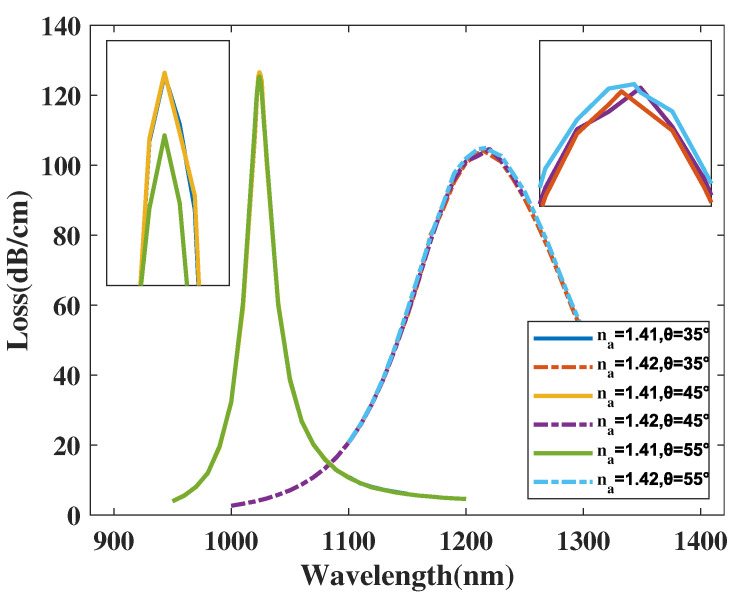
The CL curve of the *y*-polarized mode with θ chosen as 35, 45, and $55^{\circ }$, and analyte RI as 1.41 and 1.42.

Figure 7 illustrates the CL curve of the *y*-polarized mode with θ chosen as 35, 45, and $55^{\circ }$ with the analyte RI set as 1.41 and 1.42. From Figure 7, when the analyte RI is chosen as 1.41 and 1.42 with θ increasing, the $\lambda_{SPR}$ value of the CL curve almost remains unchanged and the CL peak value changes little. A reasonable explanation for this phenomenon is that these two elliptical small holes are far from the gold nanowires and *y*-polarized mode region. Therefore, the variation has an insignificant effect on mode field distributions, thus having little impact on the *y*-polarized mode and sensing performance. According to Equation (4), the maximum $S_{n}$ values achieved were 19,000, 19,600, 19,400 nm/RIU with θ set as 35, 45, and $55^{\circ }$, respectively, signifying that the RI sensitivity of the proposed structure reaches an optimal value near $\theta =45^{\circ }$. According to Equation (5), the maximum amplitude sensitivity can reach about 2220, 2226.7, and 2233.3 RIU${}^{-1}$ when θ is set as 35, 45, and $55^{\circ }$, respectively, when the analyte RI is set as 1.42. The calculation results infer that the maximum $S_{a}$ value of the proposed structure changes slightly with variation in θ, but the proposed structure always has an excellent sensing performance. In conclusion, the deviation existing in the fabrication process negligible impacts the mode field distributions and sensing performance of the proposed sensor, thus lowering the manufacturing difficulty and increases the fault tolerance in the fabrication process, further reducing the production cost.

**Figure 8:**
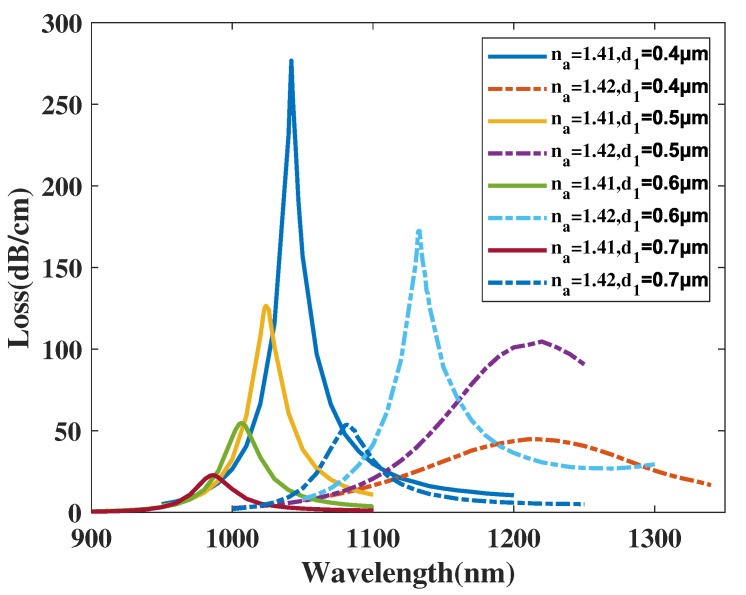
The CL curve of the *y*-polarized mode with $d_{1}$ chosen as 0.4, 0.5, 0.6, 0.7 μm, and analyte RI as 1.41 and 1.42.

Figure 8 demonstrates the CL curve of the *y*-polarized mode with $d_{1}$ chosen as 0.4, 0.5, 0.6, 0.7 μm, and analyte RI as 1.41 and 1.42. From Figure 8, when the analyte RI is chosen as 1.41 and $d_{1}$ increases from 0.4 to 0.7 μm, the $\lambda_{SPR}$ value of the CL curve exhibits a noticeable blue-shift and the CL curve peak value significantly drops. The $\lambda_{SPR}$ value of the CL curve with analyte RI set as 1.42 sees a significant blue-shift and the CL curve peak value first increases and then decreases. Furthermore, as can be seen in Figure 7, the FWHM value of the CL spectra appears to decrease significantly when $d_{1}$ increases. Furthermore, when making a comparison among the CL peak values and related envelopes, it can be deduced that when the $d_{1}$ value increases, the envelope of the CL spectra in association with the changing analyte RI appears to noticeably red-shift and then blue-shift. A reasonable explanation for this phenomenon is that when $d_{1}$ increases, the area of the *y*-polarized mode is gradually compressed, strengthening the intensity of the CL peak value. However, on the other hand, the increasing small air circles narrow the channel linking the two gold nanowires and the *y*-polarized mode area, suppressing the SPR effect and changing the mode field distributions, resulting in an intensity decline and a CL curve shift. In accordance with Equation (4), the maximum $S_{n}$ values can reach 17,200, 19,600, 12,550, and 9450 nm/RIU when $d_{1}$ is set as 0.4, 0.5, 0.6, and 0.7 μm, respectively. The calculation results illustrate that the $S_{n}$ value of the proposed structure first increases and then decreases with $d_{1}$ changing from 0.4 to 0.7 μm, reaching a maximum near $d_{1}=0.5\;$ μm. In accordance with Equation (5), the maximum $S_{a}$ values achieved were 349.9, 2226.7, 4900, and 4130.8 $RIU^{-1}$ when $d_{1}$ was set as 0.4, 0.5, 0.6, and 0.7 μm, respectively, when the analyte RI was set as 1.42. In conclusion, the amplitude sensitivity of the proposed structure first increases significantly and then declines with $d_{1}$ increasing from 0.4 to 0.7 μm. Moreover, the sensing performance is significantly improved with the variation.

**Figure 9:**
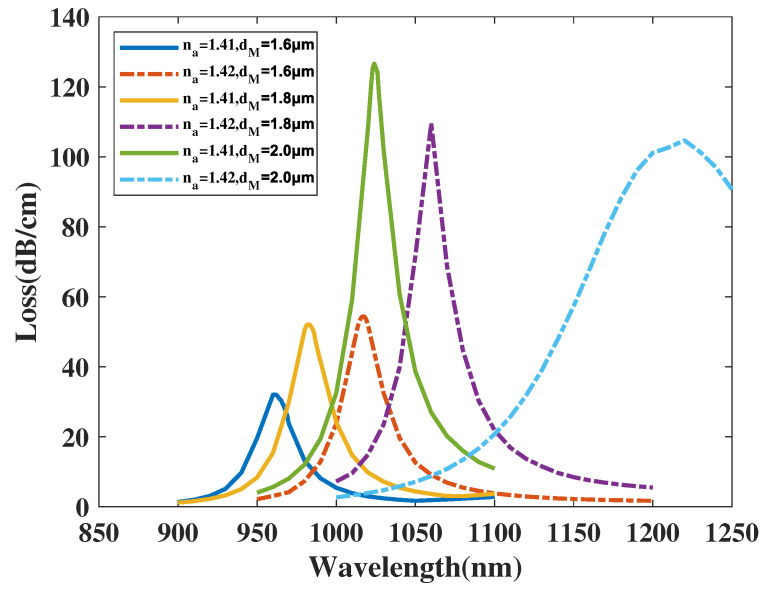
The *CL* curve of *y*-polarized mode with $d_{M}$, respectively, set as 1.6, 1.8, 2.0 μm, and analyte RI is chosen as 1.41 and 1.42.

Figure 9 illustrates the CL curve of the *y*-polarized mode with $d_{M}$ chosen as 1.6, 1.8, 2.0 μm, and analyte RI chosen as 1.41 and 1.42. From Figure 9, when $d_{M}$ increases from 1.6 to 2.0 μm, the $\lambda_{SPR}$ value of the CL curve with analyte RI chosen as 1.41 has a visible red-shift and the CL peak value has a significant boost. The resonance wavelength of the CL spectra with an analyte RI of 1.42 sees a wide-ranging red-shift and the CL curve peak value sees a noticeable growth. Furthermore, when making a comparison among the CL peak values and related envelopes, it can be found that when the $d_{M}$ value increases, the envelope of the CL spectra in correlation with the changing analyte RI appears to visibly red-shift and become wider. A reasonable explanation for this phenomenon is when the $d_{M}$ is increasing, the area of the *y*-polarized mode region is effectively compressed, greatly strengthening the intensity of the peak value. Furthermore, the increase in $d_{M}$ smooths the channel between linking the gold nanowires and the *y*-polarized area, changing the phase-matching condition, thus resulting in an intensity increase and a CL curve shift. In accordance with Equation (4), the maximum $S_{n}$ values achieved were 19,600, 17,750, 5600 nm/RIU with $d_{M}$, respectively, chosen as 1.6, 1.8, 2.0 μm. The calculation results show the $S_{n}$ value of the proposed structure witnesses a great fall with $d_{M}$ changing from 1.6 to 2.0 μm. In accordance with Equation (5), the maximum $S_{a}$ value achieves about 2226.7, 2915.96, and 1714.3 RIU${}^{-1}$ when $d_{M}$ is, respectively, chosen as 1.6, 1.8, 2.0 μm and analyte RI is set as 1.42. It’s concluded that the amplitude sensitivity of the proposed structure firstly climbs up significantly and then declines greatly with $d_{M}$ increasing from 1.6 to 2.0 μm.

**Figure 10:**
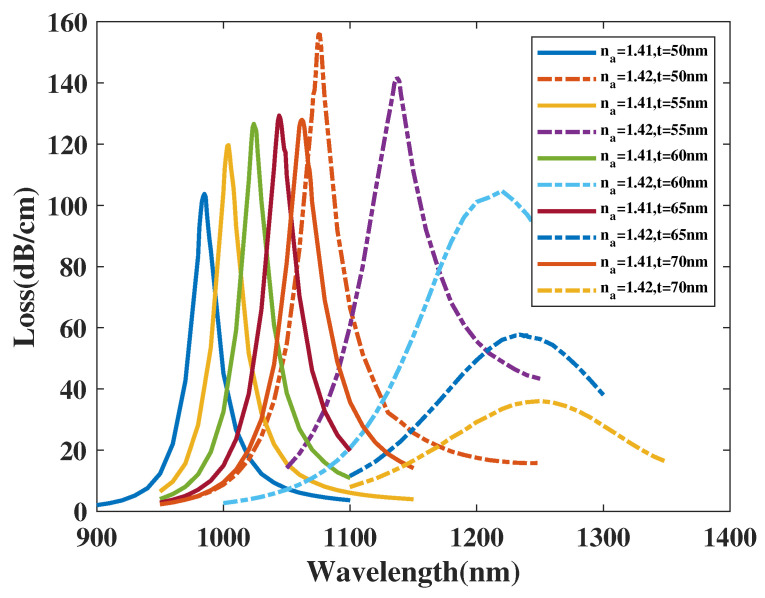
The *CL* curve of *y*-polarized mode with $t_{Au}$(abbreviated as *t*), respectively, chosen as 50, 55, 60, 65, 70 nm, and analyte RI is set as 1.41 and 1.42.

Figure 10 shows the *CL* curve of *y*-polarized mode with $t_{Au}$(abbreviated as *t*), respectively, chosen as 50, 55, 60, 65, 70 nm, and analyte RI is set as 1.41 and 1.42. From Figure 10, when analyte RI is chosen as 1.41 and *t* increases from 50 to 70 nm, the $\lambda_{SPR}$ value of the *CL* curve has a noticeable red shift and the peak value of *CL* curve firstly climbs up and then declines slightly. The resonance wavelength of the *CL* spectra with analyte RI 1.42 witness a significant red shift and the peak value of *CL* curve reduces in a large scale. Moreover, it is clear to be found that the FWHM of the *CL* spectra receives a noticeable boost with $b_{2}$ increasing when the analyte RI is set as 1.42. Furthermore, when making a comparison among those peak values of *CL* curve and related envelopes, it can be inferred that with *t* value increasing, the envelope of the *CL* spectra in company with changing analyte RI occurs to red-shift and becomes wider visibly. A reasonable explanation for the phenomenon is when *t* increases, the SPR effect is further excited, which strengthens the intensity of the peak value and alters mode field distributions, thus influence *y*-polarized mode and lead to a red shift of *CL* curve. In accordance with Equation (4), the maximum $S_{n}$ value can achieves 9050, 13,350, 19,600, 19,000, and 19,200 nm/RIU when *t* is, respectively, set as 50, 55, 60, 65, and 70 nm. The calculation results demonstrate the $S_{n}$ value of the proposed structure climbs up significantly at first and then almost remains the same and changes slightly with *t* increasing from 50 to 70 nm. In accordance with Equation (5), the maximum $S_{a}$ value achieves about 3020, 3195.3, 2226.7, 1382.1, and 555.3 RIU${}^{-1}$ when *t* is, respectively, set as 50, 55, 60, 65, and 70 nm and analyte RI set as 1.42. In summarize, the $S_{a}$ value of the proposed structure firstly rises and then declines dramatically when *t* changes from 50 to 70 nm. As a brief conclusion from Figure 10, the uprising value of thickness of gold coating causes the resulting *CL* spectrum to red shift along with a relatively stable *CL* curve intensity when analyte RI is 1.41 and a drastically attenuation of *CL* peak value when analyte RI is 1.42.

**Figure 11:**
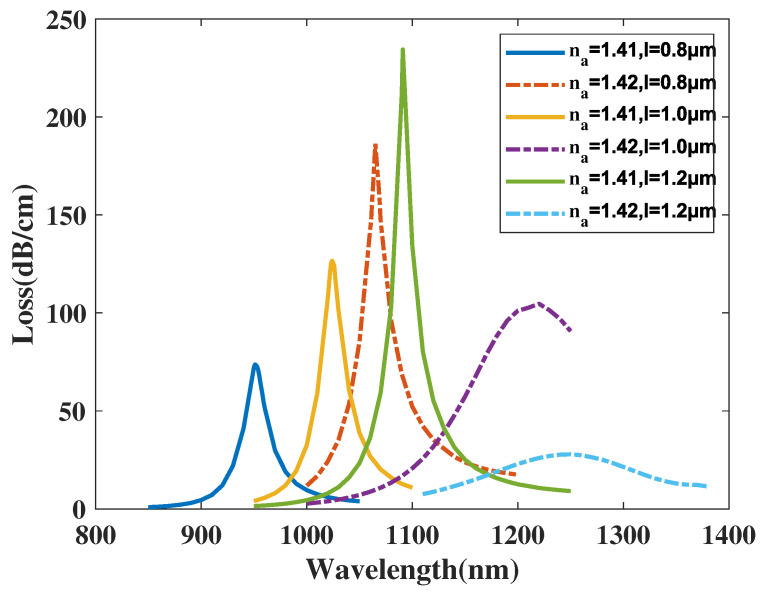
The CL curve of the *y*-polarized mode with $l_{Au}$ (abbreviated as *l*) chosen as 0.8, 1.0, 1.2 μm, and analyte RI as 1.41 and 1.42.

Figure 11 illustrates the CL curve of the *y*-polarized mode with $l_{Au}$ (abbreviated as *l*), respectively, chosen as 0.8, 1.0, 1.2 μm, and analyte RI is set as 1.41 and 1.42. From Figure 11, when analyte RI is chosen as 1.41 and *t* increases from 0.8 to 1.2 μm, the $\lambda_{SPR}$ value of *CL* spectra has a wide-ranging red shift and the peak value of *CL* curve climbs up dramatically. The $\lambda_{SPR}$ value of *CL* spectra with analyte RI 1.42 witness a wide-ranging red shift and the peak value of *CL* curve diminishes significantly. Moreover, it is obviously that the FWHM of the *CL* spectra are significantly wider with *l* increasing with analyte RI set as 1.42. Furthermore, when making a comparison among those *CL* peak values and related envelopes, it can be deduced that with *l* value increasing, the envelope of the *CL* spectra in regard to changing analyte RI emerges to red-shift. A reasonable explanation for the phenomenon is when *l* increases, the SPR effect is further excited, which strengthens the intensity of the peak value and mode field distributions, thus influence *y*-polarized mode and lead to a red shift of *CL* curve. In accordance with Equation (4), the maximum $S_{n}$ value achieves 11,100, 19,600, 18,500 nm/RIU with *l*, respectively, chosen as 0.8, 1.0, 1.2 μm. The calculation results demonstrate the $S_{n}$ value of the proposed structure firstly climbs up significantly and then decreases with *l* changing from 0.8 to 1.2 μm. In accordance with Equation (5), the maximum $S_{a}$ value achieves about 6110, 2226.7, and 206.95 RIU${}^{-1}$ when *t* is chosen as 0.8, 1.0, and 1.2 μm with analyte RI set as 1.42. In conclusion, the $S_{a}$ value of the fiber structure witnesses a dramatic fall with *l* changing from 0.8 to 1.2 μm and when *l* value is set as an appropriate small value, the proposed structure obtains an superior sensing performance. As a brief conclusion from Figure 11, the uprising value of length of gold film causes the resulting *CL* spectrum to red shift along with a dramatically boost of *CL* curve intensity when analyte RI is 1.41 and a significant fall of *CL* peak value when analyte RI is 1.42.

**Figure 12:**
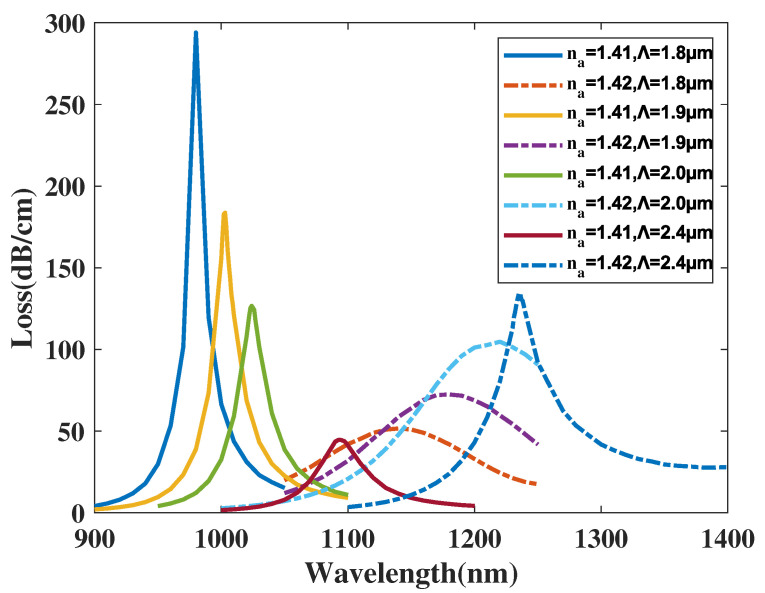
The CL curve of the *y*-polarized mode with Λ chosen as 1.8, 1.9, 2.0, 2.4 μm, and analyte RI as 1.41 and 1.42.

Figure 12 demonstrates the CL curve of the *y*-polarized mode with Λ chosen as 1.8, 1.9, 2.0, 2.4 μm, and analyte RI set as 1.41 and 1.42. From Figure 12, when Λ increases from 1.8 to 2.4 μm, the $\lambda_{SPR}$ value of the CL spectra with an analyte RI chosen as 1.41 has a visible red-shift and the CL curve peak value noticeably falls. The $\lambda_{SPR}$ value of the CL spectra with an analyte RI set as 1.42 sees a wide-ranging red-shift and the CL curve peak value gradually increasing. Furthermore, when making a comparison among the CL peak values and related envelopes, it is deduced that when Λ increases, the envelope of the CL curve in association with the changing analyte RI appears to red-shift. A reasonable explanation for this phenomenon is that with Λ increasing, the region of the *y*-polarized mode becomes compact, impacting the *y*-polarized mode and results in a CL curve shift. In accordance with Equation (4), the maximum $S_{n}$ values achieved were 16,000, 13,800, 19,600, 14,250 nm/RIU with Λ set as 1.8, 1.9, 2.0, and 2.4 μm, respectively. The calculation results illustrate that the RI sensitivity of the sensor structure first increases and then decreases with a variation in Λ from 1.8 to 2.4 μm. In accordance with Equation (5), the maximum $S_{a}$ values achieved were 931.4, 1253.7, 2226.7 and 4400 RIU${}^{-1}$ when Λ was set as 1.8, 1.9, 2.0, and 2.4 μm, respectively, when the analyte RI was set as 1.42, showing that the $S_{a}$ value of the sensor structure sharply increases when Λ changes from 1.8 to 2.4 μm, thus gaining an phenomenal sensing performance.

For the sensor structure in this work, the effects of the structural parameters on the sensing performance are summarized in the following Table 1.

## 4. Sensing Performance

After analysing the effects and sensitivity of single parameters on the sensing performance of the sensor in this work, the conclusions are as shown in Table 1.

By optimizing the parameters of the proposed structure with both $S_{n}$ and $S_{a}$ taken into consideration, we conclude with the final optimized sensor with its structural parameters: $a_{1}=1.8\;$ μm, $b_{1}=0.6\;$ μm, $a_{2}=0.8\;$ μm, $b_{2}=0.3\;$ μm, $a_{3}=0.9\;$ μm, $b_{3}=0.5\;$ μm, $t_{Au}=60$ nm, $l_{Au}=1\;$ μm, Λ=2 μm. In this section, we simulate and analyse the sensing performance of the optimized structure.

**Figure 13:**
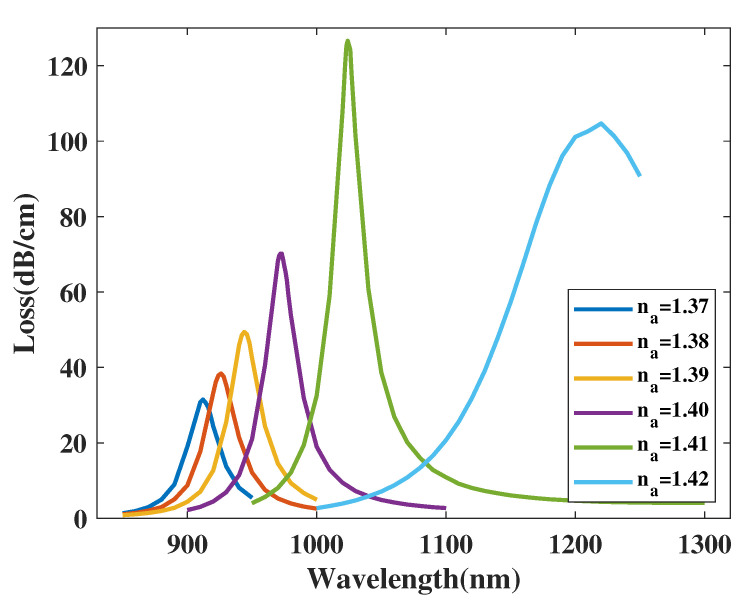
The CL curve of the y−polarized mode with a variation in the analyte RI from 1.37 to 1.42.

Figure 13 shows the CL curve of the *y*-polarized mode with variation in the analyte RI from 1.37 to 1.42. In Figure 13, when the analyte RI increases, the CL curve of the *y*-polarized mode sees an wide-ranging red-shift. Furthermore, the related CL curve peak values first increase significantly and then decrease when increasing the analyte RI. The $\lambda_{SPR}$ value shows a changing sequence: 916, 926, 944, 972.5, 1024, 1220 nm when changing the analyte RI 1.37, 1.38, 1.39, 1.40, 1.41, 1.42, respectively. In accordance with Equation (4), the related $S_{n}$ value can be calculated as 1000, 1800, 2850, 5150, and 19,600 nm/RIU, respectively. The RI coverage of the application in this work is from 1.37 to 1.42. Moreover, the average $S_{n}$ value can be calculated as 6080 nm/RIU. Thus, for the metric $S_{n}$, the structure achieved the maximum resolution of $5.102\times 10^{-6}$ RIU.

**Figure 14:**
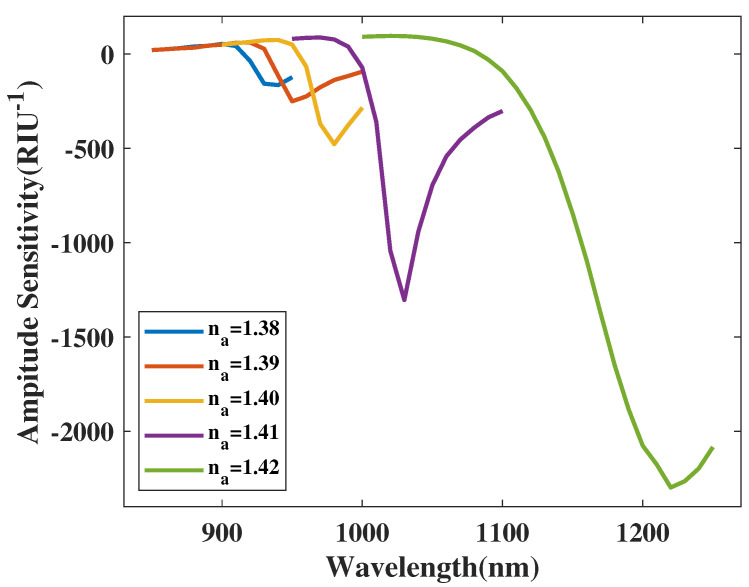
The $S_{a}$ curve of the y−polarized mode with a variation in the analyte RI from 1.38 to 1.42.

Figure 14 shows the $S_{a}$ curve of the *y*-polarized mode with a variation in the analyte RI from 1.38 to 1.42. In Figure 14, when the analyte RI increases, the CL curve of the *y*-polarized mode sees an ultra-wide-ranging red-shift. Furthermore, the related absolute value of the $S_{a}$ curve peak values dramatically boosts when increasing the analyte RI. In accordance with Equation (5), the related absolute values of $S_{a}$ can be calculated as 200, 400, 600, 1500, and 2300 RIU${}^{-1}$. The RI coverage of the application in this article is from 1.37 to 1.42. Moreover, the maximum absolute value of $S_{a}$ can be calculated as 2300 RIU${}^{-1}$. Thus, for the metric $S_{a}$, the structure achieves the maximum resolution value as $4.347826\times 10^{-6}$ RIU.

One important factor for the sensing performance of the proposed sensor structure is the coverage of the analyte RI. From Figure 13, when the RI has a measurement range from 1.37 to 1.42, the level of red-shift increases with an increase in RI. The analysis demonstrated that level of red-shift is negligible below this RI measurement coverage, dramatically decreasing the average $S_{n}$ value and prevents the proposed design to yield an ideal result. When the analyte RI is above 1.42, the SPR effect is hindered. The CL curve results from the simulation is divergent, failing to form a CL peak. Therefore, measuring coverage above 1.42 is moot. After weighing the pros and cons of both the sensing performances and measuring width, the measurement range of the RI is finally chosen as a coverage from 1.37 to 1.42.

In addition, by determining the FWHM values of each variation interval from 1.37 to 1.42 as about 30 nm, the FOM values of the proposed sensor structure can be calculated as 33.3, 60, 93.3, 171.6 and 653.3 ${\mathrm{RIU}}^{-1}$, respectively, in accordance with Equation (6). The calculation results further illustrate the proposed structure has a better performance.

In related works, coupling of the base mode and different-order SPP modes along with different polarized states have been applied to measure the analyte RI. In this work, the proposed D-shaped sensor structure is capable of measuring the analyte RI for wavelengths ranging from 850 to 1350nm, leading to its realization in the *y*-polarized mode with a negligible result in the *x*-polarization mode. Table 2 illustrates the comparison of the $S_{n}$ and absolute $S_{a}$ values of the proposed fibre structure with related works.

## 5. Conclusions

In summary, an easily manufactured D-shaped photonic crystal fibre sensor structure based on SPR the measure analyte RI is proposed. With application of two gold nanowires and *y*-polarized regions to assure SPR excitation with various physical values, the analyte RI can be measured with a wavelength coverage from 850 to 1350nm. The numerical simulation demonstrates that the maximum and average $S_{n}$ values of the proposed D-shaped fibre structure can achieve 19,600 and 6080 nm/RIU, respectively, with a RI coverage from 1.37 to 1.42 with negligible crosstalk. Moreover, with a significant red-shift in the CL spectra with an RI variation from 1.41 to 1.42, the proposed sensor structure obtains an extreme high $S_{a}$ value. The numerical simulation shows that the maximum absolute value of $S_{a}$ of the purposed design can reach $2300\;{\mathrm{RIU}}^{-1}$, further improving its sensing performance. Benefiting from these outstanding results, the purposed D-shaped SPR-based PCF sensor will have wide applications in biomedical detection and environmental science.

## Figures and Tables

**Table 1:** Effects of the structural parameters on the sensing performance of the sensor in this work.

Parameter	$\lambda_{\mathrm{SPR}}$ ^1^	Max $S_{n}$ Value	Envelope	Max $S_{a}$ Value
$a_{1}\;or\;b_{1}↑$ ^2^	Red Shift	↑↓	Blue-Shift	↓^3^
$a_{2}\;or\;b_{2}↑$	Blue-Shift	↑↓ ^4^	Red-Shift	↑
$a_{3}\;or\;b_{3}↑$	Almost Still	Almost Still	Almost Still	↓
θ↑	Almost Still	Almost Still	Almost Still	↑
$d_{1}↑$	Blue-Shift	↑↓	Blue-Shift and narrow	↑↓
$d_{M}↑$	Red-Shift	↓	Red-Shift and widen	↑↓
$t_{Au}↑$	Red-Shift	↑↓	Red-Shift and widen	↑↓
$l_{Au}↑$	Red-Shift	↑↓	Red-Shift	↓
Λ↑	Red-Shift	↑↓	Red-Shift	↑

^1^ resonant wavelength ^2^ increases. ^3^ decreases. ^4^ firstly increases and then decreases.

**Table 2:** Comparison results of the $S_{n}$ and absolute $S_{a}$ values of the proposed fibre structure with other related works.

λ	RI Coverage	$S_{n}$	$S_{a}$	Ref.
400~950	1.32~1.41	9000 (Max)	1540 (Max)	[37]
1380~2260	1.26~1.38	35,000 (Max)	1120.73 (Max)	[38]
500~950	1.33~1.44	10,300 (Max)	2245 (Max)	[39]
500~1100	1.35~1.41	20,000 (Max)	448.4 (Max)	[40]
1548~2683	1.423~1.523	12,500 (Max) 11,350 (Avg)	550 (Max)	[41]
1700~3150	1.32~1.39	40,000 (Max)	328 (Max)	[42]
850~1350	1.37~1.42	19,600 (Max) 6080 (Avg)	2300 (Max)	This work

## Data Availability

The datasets generated during the current study are available from the corresponding author on reasonable request.

## Abbreviations

The following abbreviations are used in this manuscript:

SPR	Surface plasmon resonance
PCF	Photonic crystal fibre
RI	Refractive index
WS	Wavelength sensitivity
AS	Amplitude sensitivity
FEM	Finite element method
CL	Confinement loss
